# Do Biological and Bedsite Characteristics Influence Survival of Neonatal White-Tailed Deer?

**DOI:** 10.1371/journal.pone.0119070

**Published:** 2015-03-03

**Authors:** M. Colter Chitwood, Marcus A. Lashley, John C. Kilgo, Kenneth H. Pollock, Christopher E. Moorman, Christopher S. DePerno

**Affiliations:** 1 Department of Forestry and Environmental Resources, North Carolina State University, Raleigh, North Carolina, United States of America; 2 USDA Forest Service, Southern Research Station, New Ellenton, South Carolina, United States of America; 3 Department of Applied Ecology, North Carolina State University, Raleigh, North Carolina, United States of America; University of Illinois at Urbana-Champaign, UNITED STATES

## Abstract

Coyotes recently expanded into the eastern U.S. and potentially have caused localized white-tailed deer population declines. Research has focused on quantifying coyote predation on neonates, but little research has addressed the potential influence of bedsite characteristics on survival. In 2011 and 2012, we radiocollared 65 neonates, monitored them intensively for 16 weeks, and assigned mortality causes. We used Program MARK to estimate survival to 16 weeks and included biological covariates (i.e., sex, sibling status [whether or not it had a sibling], birth weight, and Julian date of birth). Survival to 16 weeks was 0.141 (95% CI = 0.075-0.249) and the top model included only sibling status, which indicated survival was lower for neonates that had a sibling. Predation was the leading cause of mortality (35 of 55; 64%) and coyotes were responsible for the majority of depredations (30 of 35; 86%). Additionally, we relocated neonates for the first 10 days of life and measured distance to firebreak, visual obstruction, and plant diversity at bedsites. Survival of predation to 10 days (0.726; 95% CI = 0.586-0.833) was weakly associated with plant diversity at bedsites but not related to visual obstruction. Our results indicate that neonate survival was low and coyote predation was an important source of mortality, which corroborates several recent studies from the region. Additionally, we detected only weak support for bedsite cover as a covariate to neonate survival, which indicates that mitigating effects of coyote predation on neonates may be more complicated than simply managing for increased hiding cover.

## INTRODUCTION

Recent declines in white-tailed deer (*Odocoileus virginianus*) numbers, harvest, or recruitment in some areas of the southeastern U.S. (e.g., [1]) run counter to the commonly reported trend of overabundance (e.g., [2]). Though some landscapes will benefit from reduced deer numbers, the mechanisms causing the declines and the long-term trajectory of the deer populations are of interest to wildlife managers. Additionally, given the prevalence of overabundance problems, managers have attempted to limit deer population size through antlerless harvest [3], so understanding how some areas are experiencing deer population decline is important to adaptive management programs.

Coyotes (*Canis latrans*) recently expanded into the eastern U.S. [4] and have been implicated as a potential cause of localized deer population decline [5]. Coyotes are non-native additions to the southeastern U.S. landscape, having occupied the region primarily by anthropogenic means during the past 10–40 years [6, 7], with most populations established for <20 years [1]. The absence of red wolves (*Canis rufus*), modification of the landscape by humans, and merging of local coyote populations via dispersal contributed to the expansion and increased success of coyotes [6, 8].

Kilgo et al. [1] hypothesized that coyote depredation of neonatal fawns could be responsible for declining deer population metrics in South Carolina and across the region. Subsequently, Kilgo et al. [5] documented low neonatal fawn survival at their South Carolina study site and determined coyotes were the leading cause of mortality. Though studies have documented minimal predation effects of coyotes on deer in historic coyote ranges (5–17%; [9, 10, 11]), the few studies conducted in the southeastern U.S. have documented considerably greater rates of coyote predation on neonates (42%, [12]; 37–80%, [5]; 65%, [13]). Thus, the need for additional studies is great, especially those focused on the factors that contribute to or reduce predation risk for neonatal fawns. Kilgo et al. [1] posed several questions that had potential bearing on how researchers could better understand the deer-coyote dynamic in the Southeast, including how vegetation structure or other landscape variables would affect predation level. Thus, our objectives were two-fold: 1) quantify neonate survival and identify causes of mortality to determine whether coyotes were contributing to the decline of a North Carolina deer population; and 2) evaluate the effects of vegetative cover on neonate survival. We hypothesized that neonate survival would be low and coyote predation would be the leading cause of mortality. Additionally, because neonates rely on crypsis as the primary means of predator avoidance at young ages [14], we hypothesized that bedsites with greater vegetative cover would provide more protection from coyote predation and therefore be positively associated with survival.

## MATERIALS AND METHODS

### Ethics Statement

We conducted this research in accordance with the United States Department of Defense and Fort Bragg Military Installation research permit. This research required capture and handling of adult and neonate white-tailed deer, and all methods were approved by the North Carolina Wildlife Resources Commission and the North Carolina State University Institutional Animal Care and Use Committee (10–143-O).

### Site Description

We conducted our study at Fort Bragg Military Installation (hereafter, Fort Bragg), a 40,500-ha property owned by the U.S. Department of Defense and located in the Sandhills physiographic region of central North Carolina. Uplands were dominated by longleaf pine (*Pinus palustris*) forests and managed with growing-season prescribed fire on a 3-yr fire-return interval. Densely vegetated drainages were interspersed throughout the landscape. An extensive, drivable firebreak network facilitated the implementation of the large-scale fire regime, while providing access for military vehicles [15].

Deer population density was low (2–4 deer/km^2^), and hunting occurred from the first Saturday in September through 1 January in most areas of Fort Bragg. Harvest records, track counts, spotlight counts, and biologists’ observations indicated a decline in deer density from 1989 to present, commensurate with the initiation and establishment of coyotes at Fort Bragg (J. Jones, Fort Bragg Wildlife Branch, personal communication). Total hunter harvest fell from a high of 1261 in 1989 to a low of 163 in 2003 and currently averages 250–300 deer per year. Though hunter effort has changed over the years, deer hunters currently harvest deer in 1 out of 33 hunts, compared to 1 out of 15 hunts in the 1980s (J. Jones, Fort Bragg Wildlife Branch, personal communication). The first coyote at Fort Bragg was documented in 1989, and by the mid-1990s, coyotes were common. Coyote hunting is legal at Fort Bragg, but hunter effort and reported kill rates are low (i.e., a few coyotes per year); the few coyotes killed each year generally are shot opportunistically by deer hunters (J. Jones, Fort Bragg Wildlife Branch, personal communication). Coyote trapping is not allowed at Fort Bragg but is common on adjacent private land.

### Adult Female Capture and Handling

During January-May, 2011–2012, we captured females ≥ 1.5-year old using tranquilizer guns from tree stands over food plots baited with shelled corn and from vehicles. We radiocollared (Wildcell, Lotek Wireless Inc., Newmarket, Ontario, Canada; Model 2510B, Advanced Telemetry Systems, Isanti, MN), ear-tagged, and implanted each female with a vaginal implant transmitter (VIT; Model M3930, Advanced Telemetry Systems) to facilitate capture of neonates. Implantation procedures generally followed Bowman and Jacobson [16] and Carstensen et al. [17], except that we did not trim protruding antennas [5]. We used Telazol (5 mg/kg; Midwest Veterinary Supply, Burnsville, MN), xylazine hydrochloride (2.5 mg/kg; Congaree Veterinary Pharmacy, Cayce, SC), and ketamine hydrochloride (5 mg/kg; Midwest Veterinary Supply, Burnsville, MN) in 2-cc transmitter darts. At 80-minutes post-injection, we antagonized the xylazine hydrochloride with tolazoline hydrochloride (10 mg/kg; Midwest Veterinary Supply, Burnsville, MN) and monitored the deer until recovery.

### Neonate Capture and Handling

We monitored VIT signals weekly from capture until 1 May, daily until the first birth, and at 8-hour intervals (beginning at 0600, 1400, and 2200 hours) thereafter. The VITs were equipped with a thermistor that detected and signaled the change in temperature associated with expulsion of the transmitter during parturition. Additionally, VITs included a timer that indicated the number of 30-minute intervals elapsed since parturition (i.e., temperature change). We allowed ≥ 2 hours after the parturition time derived from the VIT timer before initiating a search, which provided time for grooming and initial bonding between female and neonates. Using volunteers and undergraduate students (10–40 individuals), we conducted two organized searches (~2–3 hours) during peak fawning each year. Volunteers walked transects side-by-side through all cover types. We conducted searches in areas where we had worked to capture adult females, so opportunistic captures were not biased by cover type or proximity to roads.

When we located neonates, we used latex gloves to blindfold and weigh them in a cotton bag. We estimated age of opportunistically captured neonates using new hoof growth [18] and behavior. We determined sex, deployed an expandable breakaway radiocollar ([19]; Model M4210, Advanced Telemetry Systems), and released neonates at the capture location. Radiocollars were equipped with a motion-sensitive mortality switch on a 4-hour delay.

### Fate Determination

We monitored survival of neonates remotely via VHF signal ≤ every 8 hours to 4 weeks of age, 1 to 2 times daily to 12 weeks of age, and once every 3 days until 16 weeks of age. We monitored neonates more intensively at younger ages because it has been suggested this period is when most mortality occurs [20]. Intensive monitoring allowed us to better detect mortalities, to more precisely pinpoint time of mortality, and to recover carcasses as soon as possible to reduce chance of scavenging and preserve the most evidence to be used in determining the cause of mortality [5]. When we detected a mortality signal, we proceeded immediately to recover the transmitter and remains. We efficiently accessed all of our collared neonates due to the extensive firebreak network at Fort Bragg and reached carcasses in < 30 minutes after detections, which meant the time lag between cessation of collar movement and our recovery was between 4.5 and 12.5 hours (given that a live signal could have been detected 8 hours earlier, the collar was then motionless for 4 hours prompting the mortality signal, and it took 30 minutes to reach the collar).

Following the methods of Kilgo et al. [5], we assigned initial, field-based cause of mortality based on evidence at or near the collar or remains. When sufficient remains were present to locate a killing bite wound (i.e., canine puncture wounds on the head or neck that included subcutaneous hemorrhaging [21, 22]), we assigned cause of death as predation. In these cases, we identified the predator responsible (either bobcat [*Lynx rufus*] or coyote) based on cache characteristics, tracks or scat at the recovery site, amount of remains left, parts of carcass where feeding had occurred, and location of the recovery site in relation to the neonate’s home range. Bobcats typically feed at or near the kill site [23, 24, 25], while coyotes may carry kills considerable distances (e.g., to a den or rendezvous site; [26]). Bobcats tend to cache remains under sticks, leaf litter, or debris without digging into mineral soil, while coyotes dig into mineral soil, if they cache at all [27]. Bobcats tend to focus feeding on the shoulders, while coyotes feed first on the viscera and hindquarters [27]. Additionally, coyotes are more likely to consume the entire carcass than bobcats [20, 21, 22, 24, 28]. If we were unable to recover a head or neck with killing bite wounds, but the evidence suggested the presence of a particular predator as described above, we assigned the cause of mortality as predation by that particular species (e.g., a drop of blood on vegetation adjacent to a collar with a coyote track beside it would be assigned as a coyote predation).

When no evidence of predation or emaciation was present, but the carcass was otherwise intact, we assigned the cause of death as unknown. When no evidence of predation was present but the carcass was intact and emaciated, we assigned cause of mortality as starvation. We conducted field necropsies (after training with a veterinarian at the North Carolina State University College of Veterinary Medicine) to confirm starvation as the cause of death (i.e., no milk in the digestive tract). Some researchers have removed starved neonates from their samples because of potential marking-induced abandonment. However, other research has suggested that doing so is unnecessary because the risk of marking-induced abandonment in white-tailed deer is low and omitting starved neonates can underestimate natural mortality [29, 30]. Natural abandonment (resulting in neonate starvation) is commonly reported in white-tailed deer and attributable to various causes [31], so we retained all starved neonates for analyses.

To confirm our field-based assessments of predation-related mortalities, we collected residual predator saliva for DNA identification of predator species. Following the methods of Kilgo et al. [5], we wiped 2–5 cotton swabs around killing bite wounds, near feeding sites on carcasses, on the head of the neonate, and on the radiocollar strap and housing. Unlike Kilgo et al. [5], when we determined by DNA that a predator was present at a radiocollar recovery site, even in the absence of killing bite wounds, we confidently assigned cause of mortality to that predator species. Though our monitoring schedule was intense, we acknowledge that it is possible that scavenging could have occurred before our recovery. However, in our study, we were unable to document a single scavenging event on 24 neonate carcasses that died of causes other than predation. Additionally, a 6-yr neonate survival study in South Carolina failed to document a single scavenging event on 21 carcasses that died of non-predatory causes (J. Kilgo, USDA Forest Service, unpublished data). Thus, the likelihood that scavenging could potentially bias our DNA-based predator identifications is low.

Wildlife Genetics International (WGI; Nelson, Canada) conducted the genetic analyses by extracting DNA from swab material using QIAGEN DNeasy Tissue kits (Valencia, CA). They determined the predator species present using a sequence-style species identification test focused on the 16S rRNA mitochondrial gene [32]. Additionally, when sufficient, quality coyote DNA was obtained, WGI conducted genotyping for individual identification using 17 microsatellite markers (as described in [5]). Both analyses were designed and developed previously for this type of application (for detailed molecular methods, see [5]).

### Measuring Vegetative Cover Covariates

We measured landscape covariates at neonate bedsites to determine their potential effects on neonate survival in the first 10 days of life. We focused on the first 10 days because neonates are less mobile during that period and tend to rely on crypsis to mitigate predation risk [14]. Once a neonate was radiocollared, we relocated it systematically via homing once every 24-hr period, making sure it was relocated at various times of day or night within the constraints of the monitoring schedule used to check VITs and neonate survival (described above). We checked the location of the dam and did not approach if she was in close proximity to the neonate. We approached neonates quietly to minimize disturbance and attempted to get close enough for a visual relocation (with ambient light or with the aid of a forward looking infrared radiometer [FLIR]). Once the bedsite was located, we took a GPS point and wrote a detailed description of the bedsite location and adjacent vegetation. If vegetation was too dense for a visual or FLIR relocation, we approached as close as possible and triangulated into the cover to determine the bedsite location. To minimize disturbance and reduce the risk of biasing neonate survival, we waited until all neonates were ≥2 weeks old to begin vegetation measurements (~first week of July for both years).

To quantify the vegetative structure at bedsites, we used a modified vegetation profile board [33]. We estimated percent horizontal cover from 0–2 m in 4 50-cm height categories by assigning visual obstruction on a 0–5 scale in each height category (where 0–5 represented 0%, 1–20%, 21–40%, 41–60%, 61–80%, or 81–100% coverage, respectively). We averaged the scores from all 4 height categories to derive a single cover value. We placed the board at plot center (i.e., in the bedsite) and viewed it from 1-m height, from 10 m away, along bearings of 0°, 120°, and 240°. Additionally, along each bearing, we recorded the number of plant species contributing to the horizontal cover. We determined final Nudds board scores and final plant diversity scores by taking the average of the 3 profile bearings at each bedsite and then averaging across all bedsites, producing a single value per metric per neonate. Also, we created a weighted index of visual obstruction by multiplying the final Nudds board score with the final plant diversity score for each neonate (e.g., 4.5 Nudds × 10 plants = 45). We created this metric because we thought it might provide a more accurate representation of structural complexity (e.g., some bedsites with low horizontal cover were associated with high plant diversity, while some areas with high cover values had low plant diversity). We determined distance to nearest firebreak using ArcMap 10 (Environmental Systems Research Institute, Inc., Redlands, CA) by calculating the average distance from bedsites to firebreak for each neonate.

### Statistical Analysis

We estimated survival rate to 16 weeks using known-fate modeling in Program MARK [34] and based the analysis on the known age of each neonate in weeks (i.e., we did not use a staggered entry approach; [35]). We used an information theoretic approach to draw inferences regarding *a priori* hypotheses about potential influences on neonate survival [36]. Following the methods of Kilgo et al. [5], we first assigned neonates to 2 groups based on calendar year (2011–2012) to test for within and among year temporal effects. We compared models in which survival varied by week (t), year (yr), differently among weeks between years (yr*t), linearly through time (T), or quadratically through time (T2). Next, we established a set of *a priori* candidate models that incorporated the best time trend predictor and included neonate biological characteristics (i.e., sex and birth weight [37] and Julian date of birth [38]) to test for potential effects on survival rate [36]. We imputed birth weight data for opportunistically captured neonates by randomly drawing from our distribution of values measured in that sex from that year. Additionally, we included sibling status (i.e., neonate twins were assigned a 1, while neonate singletons were assigned a 0) to model the potential effect of siblings on neonate survival rate. We imputed sibling status for opportunistically captured neonates (because we did not know their sibling status empirically) by randomly assigning a 1 or 0 based on the proportion of documented twin-sets in that year (S1 Dataset).

To evaluate the potential impacts of vegetative cover at bedsites on neonate mortality due to predation, we performed a second analysis in Program MARK. Following the procedures outlined above, we used known-fate modeling in Program MARK to estimate survival of predation to 10 days (i.e., the same time period for which we measured vegetation at bedsites). Therefore, we censored neonates that died of causes other than predation. We established a set of *a priori* candidate models based on our best time trend predictor from the first analysis and included vegetative covariates (i.e., Nudds board score, plant diversity score, weighted index of visual obstruction, and distance to firebreak; S2 Dataset).

For both analyses, we used Akaike’s Information Criterion (adjusted for small sample size; AICc) for model selection and considered our plausible models to be those ≤2.0 AICc units from the top model [36]. We used Akaike weights (*w*_*i*_) to evaluate the strength of evidence among competing models [36].

## RESULTS

We monitored 28 VITs in 2011 and 25 in 2012; 3 individuals were monitored in both years. Thus, we monitored 53 VITs in 50 individuals during the study. We captured ≥ 1 neonate from 35 of the 53 VITs (66%), and the total VIT-based sample included 59 neonates (23 in 2011 and 36 in 2012). For the 35 known births, we documented 23 twin sets, 10 singletons, 1 set of triplets, and 1 unknown litter size. For the unknown litter, we recovered 1 fawn ~20 hours after the VIT was expelled; thus, we do not know if it had a sibling. Additionally, 1 fawn from a twin set in 2012 was removed from the study because it had a foreleg caught in its radiocollar and starved. We captured 6 neonates opportunistically from unmarked females (4 in 2011 and 2 in 2012), resulting in a total sample of 65 neonates (27 in 2011 and 38 in 2012). Mean date of birth was 28 May in 2011 and 1 June in 2012. The earliest dates of birth were 12 May in 2011 and 15 May in 2012; the latest dates of birth were 23 June in 2011 and 15 June in 2012.

**Fig 1:**
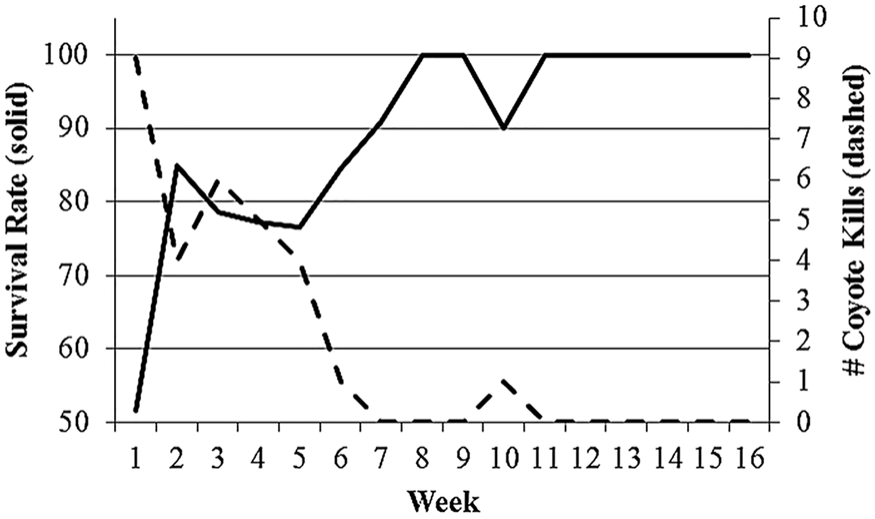
Weekly survival estimates for radiocollared neonatal white-tailed deer (solid line) and number of coyote kills per week (dashed line) at Fort Bragg Military Installation, North Carolina, 2011–2012.

Survival rates were similar across years (i.e., confidence intervals overlapped; 2011 = 0.185, 95% CI = 0.039–0.332; 2012 = 0.105, 95% CI = 0.008–0.203), so we pooled all neonates for subsequent analyses. The best model describing temporal trends in neonate survival was the *S*(t) model, and the 16-week cumulative Kaplan-Meier survival rate was 0.141 (95% CI = 0.075–0.249). Neonate survival rate was lowest during the first week of life and increased to near 1.000 around week 8 (Fig. 1).

Adding covariates to the *S*(t) model, our top model estimated survival at 0.136 (95% CI = 0.071–0.245) and included sibling status (β = -0.628, SE = 0.430, 95% CI: -1.471–0.215), indicating that survival probability was negatively associated with having a sibling. However, results should be interpreted with caution, as the β coefficient confidence interval indicated the covariate was insignificant (i.e., CI overlaps 0). Additionally, the top model did not carry much Akaike weight (*w*_i_ = 0.18), so consideration of competing models within 2 AICc was warranted (Table 1). Because no model clearly outperformed the rest, we summed Akaike weight by covariate to present the relative impact of each variable on survival (Table 2). Sibling status appeared the most in competing models, followed by Julian date of birth and sex (Table 2).

**Table 1:** Set of competing models (within 2 ΔAICc of top model) that includes biological covariates influencing neonatal white-tailed deer survival at Fort Bragg Military Installation, North Carolina, 2011–2012.

Model^a^	ΔAICc	AICw	No. parameters
*S*(t + sib)	0.0	0.181	9
*S*(t)	0.088	0.173	8
*S*(t + sib + dob)	0.237	0.161	10
*S*(t + sex)	0.755	0.124	9
*S*(t + dob)	1.658	0.079	9
*S*(t + sex + sib + dob)	1.808	0.073	11

^a^t = time effect allowed to vary weekly;

sib = sibling status; dob = Julian date of birth

**Table 2:** Summed Akaike weights (from competing models) for each biological covariate affecting neonatal white-tailed deer survival at Fort Bragg Military Installation, North Carolina, 2011–2012.

Biological Covariate^a^	Summed Akaike Weight
Sibling status (sib)	0.415
Julian date of birth (dob)	0.313
Sex (sex)	0.197

^a^Birth weight did not appear in any competing models

For the second analysis, using only the first 10 days of life to evaluate the importance of vegetative covariates on survival of predation, the *S*(t) model was again the best; it estimated survival at 0.726 (95% CI = 0.586–0.833). Adding covariates to the *S*(t) model, our top model estimated survival at 0.746 (95% CI = 0.600–0.853) and included plant diversity (β = 0.175, SE = 0.124, 95% CI = -0.069–0.419), indicating that survival probability was positively associated with bedsites with greater floral diversity. Again, results should be interpreted with caution, as the β coefficient confidence interval indicated the covariate was insignificant (i.e., CI overlaps 0). Additionally, the top model did not carry much Akaike weight (*w*_i_ = 0.18), so consideration of competing models within 2 AICc was warranted (Table 3). We summed Akaike weight by covariate to present the relative impact of each variable on survival (Table 4). Species diversity appeared the most in competing models, followed by distance to firebreak and the weighted index (Table 4).

**Table 3:** Set of competing models (within 2 ΔAICc of top model) that include vegetative covariates influencing neonatal white-tailed deer survival in the first 10 days of life at Fort Bragg Military Installation, North Carolina, 2011–2012.

Model^a^	ΔAICc	AICw	No. parameters
*S*(t + spp)	0.0	0.176	5
*S*(t)	0.102	0.168	4
*S*(t + fb + spp)	1.029	0.105	6
*S*(t + fb)	1.175	0.098	5
*S*(t + nspp)	1.220	0.096	5

^a^t = time effect allowed to vary weekly;

spp = species diversity; fb = distance to firebreak; nspp = weighted index (Nudds score × species diversity)

**Table 4:** Summed Akaike weights (from competing models) for each biological covariate affecting neonatal white-tailed deer survival in the first 10 days of life at Fort Bragg Military Installation, North Carolina, 2011–2012.

Biological Covariate^a^	Summed Akaike Weight
Species diversity (spp)	0.282
Distance to firebreak (fb)	0.204
Weighted index (nspp)^b^	0.096

^a^Nudds score did not appear in any competing models

^b^Weighted index = Nudds score × species diversity

Predation was the cause of death for 35 of the 55 neonates that died (Table 5). Based on field methods, we assigned a predator species to 35 cases and submitted swabs from all 35 (15 in 2011, 20 in 2012). Mitochondrial DNA testing successfully identified predator species for swabs from 32 of the 35 neonates (91%; 14 of 15 in 2011 and 18 of 20 in 2012). In all 3 cases in which predator DNA was not detected, field evidence was consistent with other depredations, allowing us to confidently assign predator species without DNA confirmation. We never detected mtDNA from more than one predator species at the same carcass.

**Table 5:** Causes of mortality among radiocollared neonatal white-tailed deer at Fort Bragg Military Installation, North Carolina, 2011–2012.

Cause of Mortality	2011		2012		Total	
	n	%	n	%	n	%
Coyote predation	12	54.5	18	54.5	30	54.5
Starvation	5	22.7	11	33.3	16	29.1
Bobcat predation	3	13.6	2	6.1	5	9.1
Unknown^a^	2	9.1	1	3.0	3	5.5
Vehicle	0	0.0	1	3.0	1	1.8

^a^Includes non-depredated, non-starved neonates

**Fig 2:**
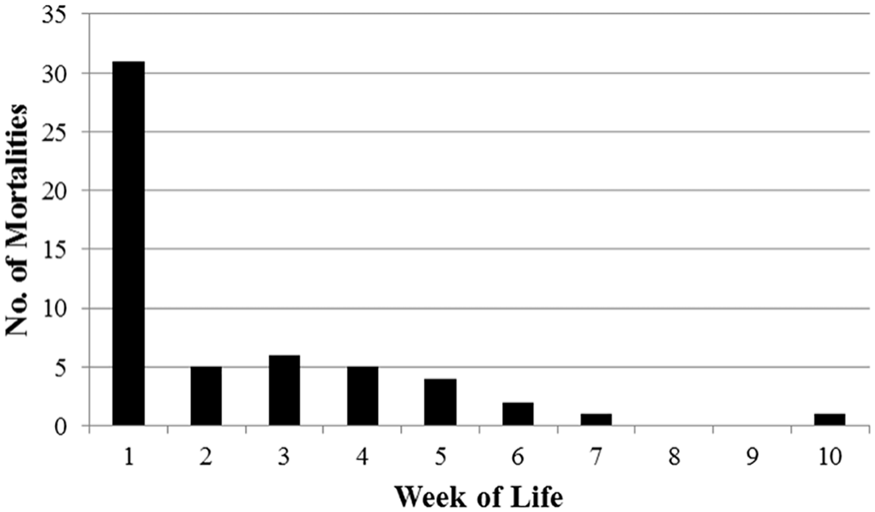
Number of mortalities by week of life among radiocollared neonatal white-tailed deer at Fort Bragg Military Installation, North Carolina, 2011–2012.

Predation by coyotes was the most frequent cause of mortality, accounting for 30 of the 55 deaths (55%; Table 5). Bobcats accounted for 5 of 55 deaths (9%; Table 5). Overall, neonate mortality was greatest during the first week of life (Fig. 2), with the latest coyote and bobcat depredations occurring in the tenth and seventh weeks of life, respectively. Starvation was the second-leading cause of mortality and accounted for 16 of 55 deaths (29%; Table 5). All neonates that died of starvation were within the first week of life (Fig. 2).

Among coyote depredations linked to coyotes by mtDNA (n = 28), sufficient DNA was obtained from 12 cases (5 in 2011, 7 in 2012) for individual coyote genotyping. Most neonates were killed or consumed by different coyotes, and we obtained 9 unique genotypes across the 12 cases. Two coyotes were detected at 2 neonates each in 2012, and 1 coyote was detected on 1 neonate in both years.

## DISCUSSION

The neonate survival rate of 14% at Fort Bragg was low relative to other studies of neonate survival in the presence of coyotes. In the western and northeastern regions of the U.S., coyotes have been implicated as the primary source of mortality, and many of those studies reported comparably low survival rates (28%, [20]; 12%, [22]; 10%, [39]; 26%, [40]). Interestingly, other studies conducted in the presence of coyotes have documented much greater survival rates (84%, [10]; 87%, [11]; 91%, [41]). Nevertheless, the few studies conducted in the southeastern U.S. where coyotes are novel predators reported low survival (22%, [5]; 33%, [12]; 26%, [13]), with coyotes as the leading cause of mortality.

Neonate independence is a topic of interest in survival studies due to the potential bias associated with including both individuals from a set of twins. Interestingly, we detected a small effect of sibling status on neonate survival based on the top model. The β coefficient was insignificant but suggested there was a slight reduction in survival for neonates having a sibling. Ecologically, this indicates that twin sets may attract more attention from predators like coyotes even if they are spatially separated. We speculate that coyotes could use behavioral cues from the dam to increase searching efficiency and perhaps benefit from twins bedded in relatively close proximity. Our results may lend support to the statistical argument that individuals from twin sets are dependent on one another and cannot both be included. However, leaving a twin uncollared or removing it randomly from later analyses does not address the dependency that exists on the landscape. The twins are still reliant upon the dam for milk, and though they are most often spatially separated, they are still dependent on the dam’s attention. Our approach allows us to include the potential twin-effect in survival studies, while maintaining sample size (i.e., knowing the fate of entire litters). To determine neonate survival and recruitment into the population, collaring the greatest number of neonates possible provides the most biological information.

Kilgo et al. [1] suggested many factors could be responsible for the magnitude of effect coyotes have on neonate survival, including coyote density, deer density, alternative coyote food sources, and vegetative hiding cover. Similar to Kilgo et al. [5], the low deer density (2–4 deer/km^2^) relative to the apparent prevalence of coyotes at Fort Bragg may explain the low rate of neonate survival in our study. Though we do not have quantitative density estimates for coyotes at Fort Bragg, anecdotal evidence indicates that coyotes are common and may exist at moderate-to-high densities relative to other places in the region. Currently, reasons for potentially high coyote density are unknown but may relate to the availability of other foods. At Fort Bragg, neonates were most susceptible to coyotes at young ages, which might indicate that coyotes switch to other food items as neonates age and become more difficult to catch [5]. Additionally, we did not detect a strong effect of date of birth on neonate survival. Numerous studies with high rates of predation on neonatal ungulates have reported no effect of birth date on survival [12, 42, 43, 44]. Thus, we suspect coyotes are not satiated by the number of neonates available during the fawning season at Fort Bragg, which was consistent with the conclusions of Kilgo et al. [5] in South Carolina. Other than density related interactions, vegetative cover seems to be a likely factor for explaining neonate survival. Unfortunately, with such a high rate of mortality as we report, it is difficult to conclude that vegetation has much effect on survival at Fort Bragg. Our best model explaining survival to 10 days included plant diversity, but the support for the model was weak and the β coefficient was insignificant. Though managers may wish to promote improvements in cover as a strategy to mitigate coyote effects on fawn recruitment, the relative ratio of coyotes to deer may be more important and overwhelm any impact of improved vegetative cover. Based on our data, the diversity of flora at neonate bedsites is more important than horizontal cover. However, lack of support for cover in explaining neonate survival is not surprising, as Kilgo et al. [45] failed to detect home range-scale effects of vegetative cover on neonate survival.

Starvation potentially resulting from abandonment by the dam was our second-leading cause of mortality (29%) and was greater than rates reported in other studies (8%, [5]; 4%, [11]; 25%, [12]; 10%, [44]; 0%, [46]). Though it is possible that capture and handling caused abandonment, we documented 6 sets of twins in which only 1 starved. Because we handled all neonates similarly, we do not believe capture-induced abandonment was an issue. More likely, predation risk could have indirect consequences for adult females. For example, studies with other ungulates have demonstrated that predation risk can negatively impact reproductive rate [47]. Further, Lashley et al. [48] demonstrated that white-tailed deer females with young decreased feeding rates at baited camera sites by almost 50%, which could suppress lactation potential via reduced foraging efficiency. Aside from indirect effects of predation, heat stress may exacerbate rates of abandonment. In 2011, the starvations (n = 5) we documented occurred during a week-long period associated with a heat wave. We speculate that heat stress in the dam could have contributed to reduced milk production, which has been demonstrated in other ruminants (e.g., cattle; [49]). Given the predicted weather-related changes under various climate change scenarios, future research should explore the potential for climate-related variables to affect the body condition or milk production of the mother and subsequent survival of her offspring.

Kilgo et al. [45] concluded that coyote predation on South Carolina neonates was an additive source of mortality. Although our study was not designed to determine whether or not coyote predation was compensatory or additive, we documented 5 cases where neonates were vocalizing as we approached to check survival or relocate the neonate and all 5 neonates subsequently starved [50]. As discussed by Chitwood et al. [50], it is possible that increased vocalization due to abandonment could predispose a neonate to coyote predation, thereby inflating the apparent role of coyote depredation. Thus, confusion in assigning ultimate cause of mortality could mask a potentially compensatory effect. However, our data do not allow us to make such distinctions, and in fact, similar to Kilgo et al. [5], we failed to document a single case in which a depredated neonate was emaciated.

When deer population objectives can be met, neonatal mortality (whether it is additive or compensatory) that occurs before the hunting season (i.e., time of recruitment) is not important [51]. However, in areas of low deer density (like Fort Bragg) where coyotes take a notable proportion of neonates (this study) and represent a source of adult deer mortality [52], managers may struggle to meet deer population objectives. In such cases, understanding additive/compensatory issues will be necessary for enacting effective management strategies (e.g., coyote removal, female deer harvest reductions) to mitigate population declines. For example, if coyote predation is an additive mortality source for neonates in the southeastern U.S. (e.g., [45]), then management actions that reduce mortality from coyotes could be effective at improving recruitment. Thus, future research should evaluate how managers may have to adapt deer management plans to meet population objectives, or adapt the objectives themselves, now that coyotes are part of the landscape.

## CONCLUSIONS

Evidence is mounting that coyotes are capable of affecting deer populations across the southeastern U.S., at least in localized areas. Our low neonate survival helps explain the apparent deer population decline documented by Fort Bragg during the establishment of the coyote population. Coupled with coyote predation on adult females documented at Fort Bragg [52], the potential impact of coyote predation on managing deer populations is great. However, the possible range of effects that coyote predation can have on deer vital rates and behaviors is unknown, so future studies need to document how coyotes impact deer in areas with greater deer density or lower coyote density. Future research should explore deer population models under various scenarios in an adaptive management framework to provide more insight into how deer populations respond to the influence of coyotes and hunter harvest (e.g., [53, 54]). In areas where deer density reduction is needed, coyotes will be an asset for managers. Conversely, harvest reductions on the female segment of the deer population may be required to offset impacts of coyote predation, particularly in areas with deer densities below target or with unsustainably low fawn recruitment. Finally, managers should focus on density issues first because vegetative cover at neonate bedsites may not provide a buffer against the impacts of coyote predation.

## Supporting Information

S1 Dataset White-tailed deer fawn encounter histories and biological covariates used for 16-week survival analysis, Fort Bragg Military Installation, North Carolina, 2011–2012. (TXT)

S2 Dataset White-tailed deer fawn encounter histories and bedsite covariates used for 10-day survival analysis, Fort Bragg Military Installation, North Carolina, 2011–2012. (TXT)

## References

[1] Kilgo JC, Ray HS, Ruth C, Miller KV. Can coyotes affect deer populations in southeastern North America? J Wildl Mgt. 2010;74: 929–933.

[2] Warren RJ. The challenge of deer overabundance in the 21st century. Wildl Soc Bull. 1997;25: 213–214.

[3] Miller KV, Marchinton RL. Quality whitetails: the how and why of Quality Deer Management. Mechanicsburg: Stackpole Books; 1995.

[4] Parker GR. Eastern coyote: the story of its success. Halifax: Nimbus Publishing; 1995.

[5] Kilgo JC, Ray HS, Vukovich M, Goode MJ, Ruth C. Predation by coyotes on white-tailed deer neonates in South Carolina. J Wildl Mgt. 2012;76: 1420–1430.

[6] Hill EP, Sumner PW, Wooding JB. Human influences on range expansion of coyotes in the southeast. Wildl Soc Bull. 1987;15: 521–524.

[7] Gompper ME. Top carnivores in the suburbs? Ecological and conservation issues raised by colonization of north-eastern North America by coyotes. Bioscience. 2002;52: 185–190.

[8] Nowak RM. North American Quaternary Canis. Monograph of the Museum of Natural History. University of Kansas. 1979;6: 1–154.

[9] Heugel CN, Dahlgren RB, Gladfelter HL. Mortality of white-tailed deer fawns in south-central Iowa. J Wildl Mgt. 1985;49: 377–380.

[10] Brinkman TJ, Jenks JA, DePerno CS, Haroldson BS, Osborn RG. Survival of white-tailed deer in an intensively farmed region of Minnesota. Wildl Soc Bull. 2004;32: 726–731.

[11] Grovenburg TW, Swanson CC, Jacques CN, Klaver RW, Brinkman TJ, Burris BM, et al Survival of white-tailed deer neonates in Minnesota and South Dakota. J Wildl Mgt. 2011;75: 213–220.

[12] Saalfeld ST, Ditchkoff SS. Survival of neonatal white-tailed deer in an exurban population. J Wildl Mgt. 2007;71: 940–944.

[13] Jackson AM, Ditchkoff SS. Survival estimates of white-tailed deer fawns at Fort Rucker, Alabama. Am Midl Nat. 2013;170: 184–190.

[14] DeYoung RW, Miller KV. White-tailed deer behavior In: Hewitt DG, editor. Biology and management of white-tailed deer. Boca Raton: CRC Press; 2011 pp. 311–351.

[15] Lashley MA, Chitwood MC, Prince A, Elfelt MB, Kilburg EL, DePerno CS, et al Subtle effects of a managed fire regime: a case study in the longleaf pine ecosystem. Ecol Indic. 2014;38: 212–217.

[16] Bowman JL, Jacobson HA. An improved vaginal-implant transmitter for locating white-tailed deer birth sites and fawns. Wildl Soc Bull. 1998;26: 295–298.

[17] Carstensen M, DelGiudice GD, Sampson BA. Using doe behavior and vaginal-implant transmitters to capture neonate white-tailed deer in north-central Minnesota. Wildl Soc Bull. 2003;31: 634–641.

[18] Sams MG, Lochmiller RL, Hellgren EC, Warde WD, Varner LW. Morphometric predictors of neonatal age for white-tailed deer. Wildl Soc Bull. 1996;24: 53–57.

[19] Diefenbach DR, Kochanny CO, Vreeland JK, Wallingford BD. Evaluation of an expandable, breakaway radiocollar for white-tailed deer fawns. Wildl Soc Bull. 2003;31: 756–761.

[20] Cook RS, White M, Trainer DO, Glazener WC. Mortality of young white-tailed deer fawns in south Texas. J Wildl Mgt. 1971;35: 47–56.

[21] White M. Description of remains of deer fawns killed by coyotes. J of Mammal. 1973; 54: 291–293.

[22] Garner GW, Morrison JA, Lewis JC. Mortality of white-tailed deer fawns in the Wichita Mountains, Oklahoma. Proc Ann Conf Southeast Assoc of Fish and Wildl Agen. 1976;30: 493–506.

[23] Beale DM, Smith AD. Mortality of pronghorn antelope fawns in western Utah. J Wildl Mgt. 1973;37: 343–352.

[24] Labisky RF, Boulay MC. Behaviors of bobcats preying on white-tailed deer in the Everglades. Am Midl Nat. 1998;139: 275–281.

[25] Roberts SB. Ecology of white-tailed deer and bobcats on Kiawah Island, South Carolina: implications for suburban habitat preservation. Ph.D. Dissertation, University of Georgia. 2007.

[26] Harrison DJ, Gilbert JR. Denning ecology and movements of coyotes in Maine during pup rearing. J of Mammal. 1985;66: 712–719.

[27] O’Gara BW. Differential characteristics of predator kills. Proceedings of the Biennial Pronghorn Antelope Workshop. 1978;8: 380–393.

[28] Epstein MB, Feldhamer GA, Joyner RL. Predation on white-tailed deer fawns by bobcats, foxes, and alligators: predator assessment. Proc Ann Conf Southeast Assoc of Fish and Wildl Agen. 1983;37: 161–172.

[29] Ozoga JJ, Clute RK. Mortality rates of marked and unmarked fawns. J Wildl Mgt. 1988;52: 549–551.

[30] Carstensen Powell M, DelGiudice GD, Sampson BA. Low risk of marking-induced abandonment in free-ranging white-tailed deer neonates. Wildl Soc Bull. 2005;33: 643–655.

[31] Langenau EE Jr., Lerg JM. The effects of winter nutritional stress on maternal and neonatal behavior in penned white-tailed deer. Appl Anim Ethol. 1976;2: 207–223. 10.1186/1297-9686-8-2-207 22896489 PMC2764577

[32] Johnson WE, O’Brien SJ. Phylogenetic reconstruction of the Felidae using 16S rRNA and NADH-5 mitochondrial genes. J Mol Evol. 1997;44: S98–S116. 9071018 10.1007/pl00000060

[33] Nudds TD. Quantifying the vegetative structure of wildlife cover. Wildl Soc Bull. 1977;5: 113–117.

[34] White GC, Burnham KP. Program MARK—survival estimation from populations of marked animals. Bird Study. 1999;46: S120–S139.

[35] Bishop CJ, White GC, Lukacs PM. Evaluating dependence among mule deer siblings in fetal and neonatal survival analysis. J Wildl Mgt. 2008;72: 1085–1093.

[36] Burnham KP, Anderson DR. Model selection and multimodel inference: a practical information—theoretic approach. New York: Springer; 2002

[37] Rohm JH, Nielsen CK, Woolf A. Survival of white-tailed deer fawns in southern Illinois. J Wildl Mgt. 2007;71: 851–860.

[38] Bishop CJ, White GC, Freddy DJ, Watkins BE, Stephenson TR. Effect of enhanced nutrition on mule deer population rate of change. Wildl Mono. 2009;172: 1–28.

[39] Bartush WS, Lewis JC. Mortality of white-tailed deer fawns in the Wichita Mountains. Proc Okla Acad Sci. 1981;61: 23–27.

[40] Long RA, O’Connell AF Jr., Harrison DJ. Mortality and survival of white-tailed deer *Odocoileus virginianus* fawns on a north Atlantic coastal island. Wildl Biol. 1998;4: 237–247.

[41] Pusateri Burroughs J, Campa H III, Winterstein SR, Rudolph BA, Moritz WE. Cause-specific mortality and survival of white-tailed deer fawns in southwestern lower Michigan. J Wildl Mgt. 2006;70: 743–751.

[42] Fairbanks WS. Birthdate, birthweight, and survival in pronghorn fawns. J of Mammal. 1993;74: 129–135.

[43] Smith BL, Anderson SH. Juvenile survival and population regulation of the Jackson elk herd. J Wildl Mgt. 1998;63: 1036–1045.

[44] Vreeland JK, Diefenbach DR, Wallingford BD. Survival rates, mortality causes, and habitats of Pennsylvania white-tailed deer fawns. Wildl Soc Bull. 2004;32: 542–553.

[45] Kilgo JC, Vukovich M, Ray HS, Shaw CE, Ruth C. Coyote removal, understory cover, and survival of white-tailed deer neonates. J Wildl Mgt. 2014;78: 1261–1271.

[46] Ballard WB, Whitlaw HA, Young SJ, Jenkins RA, Forbes GJ. Predation and survival of white-tailed deer fawns in northcentral New Brunswick. J Wildl Mgt. 1999;63: 574–579.

[47] Creel S, Christianson D, Liley S, Winnie JA Jr.. Predation risk affects reproductive physiology and demography of elk. Science. 2007;315: 960–960. 17303746 10.1126/science.1135918

[48] Lashley MA, Chitwood MC, Biggerstaff MT, Morina DL, Moorman CE, DePerno CS. White-tailed deer vigilance: the influence of social and environmental factors. PLoS ONE. 2014;9:e90652 10.1371/journal.pone.0090652 24599090 PMC3945222

[49] Rhoads ML, Rhoads RP, VanBaale MJ, Collier RJ, Sanders SR, Weber WJ, et al Effects of heat stress and plane of nutrition on lactating Holstein cows: I. Production, metabolism, and aspects of circulating somatotropin. J Dairy Sci. 2009;92: 1986–1997. 10.3168/jds.2008-1641 19389956

[50] Chitwood MC, Lashley MA, Moorman CE, DePerno CS. Vocalization observed in starving white-tailed deer neonates. Southeast Nat. 2014;13: N6–N8.

[51] Rosenberry CS, Norton AS, Diefenbach DR, Fleegle JT, Wallingford BD. White-tailed deer age ratios as herd management and predator impact measures in Pennsylvania. Wildl Soc Bull. 2011;35: 461–468.

[52] Chitwood MC, Lashley MA, Moorman CE, DePerno CS. Confirmation of coyote predation on adult female white-tailed deer in the southeastern United States. Southeast Nat. 2014;13: N30–N32.

[53] Robinson KF, Diefenbach DR, Fuller AK, Hurst JE, Rosenberry CS. Can managers compensate for coyote predation of white-tailed deer? J Wildl Mgt. 2014;78: 571–579.

[54] Chitwood MC, Lashley MA, Kilgo JC, Moorman CE, DePerno CS. White-tailed deer population dynamics and adult female survival in the presence of a novel predator. J Wildl Mgt. 2015;79: 211–219.

